# Understanding Sexual Aggression in UK Male University Students: An Empirical Assessment of Prevalence and Psychological Risk Factors

**DOI:** 10.1177/10790632211051682

**Published:** 2021-10-27

**Authors:** Samuel T. Hales, Theresa A. Gannon

**Affiliations:** 1 2240 University of Kent, Canterbury, UK

**Keywords:** campus sexual assault, college students, harm prevention, perpetration, sexual offending

## Abstract

University-based sexual aggression is an international public health issue; however, to date, there have been no formal assessments of the prevalence or psychological indicators associated with the proabuse behaviors of the most common perpetrators at UK universities: heterosexual male students. To facilitate the development of effective primary prevention interventions for domestic students who have sexually harmed, we assess across two empirical studies (*N*s = 259 and 295) the psychological risk factors associated with recent sexual aggression amongst two distinct samples of UK male university students. Cumulatively, results highlighted that one in nine participants (11.4%) self-reported recent sexual aggression. These participants could be statistically differentiated from their non-offending peers on various established indicators of general sexual offending, of which logistic regression analyses highlighted atypical sexual fantasies, general aggression, hostility toward women, and rape myth acceptance as being the most reliable predictors. Our data extend the international evidence base by providing the first detailed overview of sexual aggression amongst UK male university students, as well as the psychological risk factors associated with their proabuse behaviors. We discuss the importance of our findings for the development of more effective evidence-based reduction strategies and primary prevention interventions for male students who have sexually harmed.

## Introduction

Male sexual aggression is an international public health issue that plagues universities. Defined in this paper as a continuum of illicit behaviors characterized by any unwanted or non-consensual sexual activity, it is estimated that at least one-in-five female university students across most developed countries will be a victim of a sexually aggressive act during their studies (e.g., Association of American Universities [AAU], 2019; Krahé & Berger, 2013; Muehlenhard et al., 2017). These criminal offenses are associated with adverse long-term physical, psychological, and economic outcomes (see Brown et al., 2009), with US estimates that rape costs victims $122,461 during their lifetime (Peterson et al., 2017).

In the UK, recent national climate surveys have found that over a quarter of female students self-report sexual aggression victimization at university (National Union of Students [NUS], 2011), with eight percent declaring that they were raped (Revolt Sexual Assault, 2018). Consistent with international findings (e.g., Association of American Universities [AAU], 2019; Krahé & Berger, 2013; Schuster & Krahé, 2019), these surveys highlight that perpetrators are often known male students studying at their victim’s university (National Union of Students [NUS], 2011; Revolt Sexual Assault, 2018). Though difficult to directly compare, the prevalence of university-based sexual aggression in the UK appears notably higher than sexual aggression within the wider community, where 3.4% of females are victimized annually (Office for National Statistics, 2018).

Despite its frequency, there have been no formal estimates of the prevalence of university-based sexual aggression perpetration in the UK, nor any empirical assessments of the risk factors associated with students’ proabuse behaviors (see Jones et al., 2020). This is surprising given our established understanding of student perpetrators of sexual aggression in other countries (e.g., Abbey & McAuslan, 2004; D’Abreu & Krahé, 2014; Salazar et al., 2018; Tomaszewska & Krahé, 2018), as well as incarcerated individuals who have perpetrated sexual aggression across the wider community (e.g., Fisher et al., 1999; Hanson & Bussière, 1998; Hanson & Morton-Bourgon, 2005; Mann et al., 2010). This deficit in academic knowledge has severely hampered the development of effective evidence-based harm prevention interventions for “at-risk” male students on UK campuses, threatening the safety of female students. Of the available data in the UK, most have been collected through interviews and self-reports with victims and are limited to demographic descriptions of perpetrators only (e.g., National Union of Students [NUS], 2011; Revolt Sexual Assault, 2018). Though useful for suspect identification, this information fails to explain the risk factors that predispose male students to sexual aggression; consequently, there is no sound evidence base on which universities can develop effective interventions to reduce the high perpetration rates on campuses in the UK.

Compared to the UK, other countries have notably more developed research agendas relevant to university-based sexual aggression. In the US, for example, there has been an expanding knowledge base in the area since the mid-1950s, such that researchers there now possess a good understanding of the risk-relevant factors associated with male students’ sexual aggression, as well as their characteristics as a group of forensic interest. However, differences in the university history, geography, and culture between the US and UK (e.g., an increased emphasis in the US on alcohol consumption, fraternity membership, and sports participation amongst male university students; see Murnen & Kohlman, 2007) limit the generalizability of these findings to UK student populations (see Phipps & Smith, 2012; Stenning et al., 2012). Moreover, US research often considers university-based sexual aggression as distinct from sexual aggression committed by incarcerated persons and thus does not utilize a broad and established knowledge base already available on the topic. This includes, for example, research into the psychosocial variables associated with perpetration, which is notably absent in current US campus sexual assault literature. Adopting a more forensic psychological lens is likely to further academic understanding regarding the psychological characteristics of sexually aggressive male university students by highlighting new factors linked to their offending behaviors, in turn providing better foundations for effective harm prevention. Such an approach was recently encouraged by O’Connor et al. (2021) in their comprehensive systematic review of campus sexual violence research as a way to refine our understanding of perpetrators and their motivations toward offending.

Our studies are the first to assess empirically the psychological characteristics of sexually aggressive male university students in the UK, as well as their self-reported prevalence of sexual aggression. We extend previous international research by examining the combined predictive value of multiple psychological variables that have either been associated with convicted sexual offenses but not applied to university-based sexual aggression, or university-based sexual aggression but typically as a standalone or dual predictor only. Below, we review past literature relating to sexual aggression amongst university students in other countries before describing our current studies.

### Empirical Work Examining the Characteristics of University-based Sexual Aggression

Campus sexual assault research in other countries has demonstrated that there are specific psychological predictors of sexual aggression amongst male students (Abbey & McAuslan, 2004; D’Abreu & Krahé, 2014; Gidycz et al., 2007; Thompson et al., 2013; Tomaszewska & Krahé, 2018). These “individual-level” indicators can be divided into attitudinal, personality, and experiential risk factors (Abbey et al., 2001; Dills et al., 2016). Empirical studies in this area typically adopt a between-groups design to assess differences in scores on psychological measures between perpetrators of sexual harm and non-perpetrators and use predictive modelling procedures to establish how well they predict past sexual aggression (e.g., Gidycz et al., 2007; Salazar et al., 2018). These studies have shown that risk factors often coalesce and interact with other risk and protective factors to encourage or inhibit sexual aggression (e.g., Malamuth et al., 2021; Martín et al., 2005).

A notable body of US work suggests that male university students’ sexual aggression can be explained by their negative views about women, which result from traditional gender role socialization (see Vogel, 2000). The confluence model (Malamuth et al., 2021)—the leading integrative theoretical model of sexual aggression—proposes that this “hostile masculinity” is a key risk factor for males and increases their propensity to engage in sexual aggression. US studies have highlighted strong links between sexually aggressive behaviors in male university students and typical indices of hostile masculinity, including rape myth acceptance and hostility toward women (e.g., Abbey et al., 2001; Vogel, 2000), as well as atypical sexual fantasies that center on coercive, controlling, or illicit sexual behaviors (e.g., raping a person; Greendlinger & Byrne, 1987). These findings have been validated by researchers in other countries (e.g., Chan, 2021; Čvek & Junaković, 2020; Martín et al., 2005; Tomaszewska & Krahé, 2018), suggesting that hostile masculinity constitutes a strong predictor of sexual aggression across male student groups globally.

There is also strong support internationally for the prognostic value of less gendered attitudinal factors in predicting university-based sexual aggression, such as low self-esteem (e.g., Good et al., 1995; Schuster & Krahé, 2019), deficits in emotion regulation (e.g., Pickett et al., 2016), and aggression (e.g., Rapaport & Burkhart, 1984). These factors have also been identified as key correlates of sexually aggressive behaviors among incarcerated males (see Hanson & Bussière, 1998; Hanson & Morton-Bourgon, 2005; Mann et al., 2010), such to the extent that they form central elements of established theories of general sexual offending (e.g., Marshall & Barbaree, 1990; Ward & Beech, 2006). To date, though, no empirical research has considered their combined ability to predict sexual aggression with male university students, thus limiting the development of effective harm prevention interventions.

Other established risk factors that have been linked to sexually aggressive behaviors amongst incarcerated males, but which have not been explored extensively as predictors of university-based sexual aggression, include assertiveness and self-efficacy, especially in romantic relationships (see Fisher et al., 1999; Marshall et al., 1997; Seto & Lalumière, 2010), as well as loneliness (Ward & Beech, 2006). Researchers have proposed that these intimacy and social functioning deficits represent critical risk factors for males who have sexually harmed, who often lack meaningful interpersonal relationships, possess attachment issues, and report unfulfilling past romantic relationships (see Marshall, 2010). Assessing the prognostic value of psychosocial variables such as these could help refine academic understanding of the psychological characteristics of sexually aggressive male university students, as well as the etiology and maintenance of their offending behaviors.

Of course, it is worth noting that not all male university students are susceptible to the diathesis of sexual aggression, nor do those who display a proclivity always act on their urges (see Abbey & McAuslan, 2004). It is believed that this is because there is a developmental sequence for sexual aggression, in which personality characteristics and experiential factors establish a precondition for sexual aggression, which are then liberated in the presence of specific situational variables (see Abbey et al., 2001). Undoubtedly, the most studied situational variable relevant to university-based sexual aggression is alcohol consumption, which has been shown to significantly increase risk (for a review, see Abbey, 2002; Chan, 2021; Čvek & Junaković, 2020). Other key factors include sports participation and fraternity membership (for a review, see Murnen & Kohlman, 2007), though we speculate these may be more prominent in countries that adopt a US approach to higher education (e.g., those that have a Greek Life or collegiate sports system) versus a UK approach (where sports are typically played intramurally and fraternities do not exist).

### Purpose of the Present Studies

Despite a broad knowledge base in other countries, our literature review underlines the lack of empirical research assessing the psychological characteristics of UK male students who perpetrate university-based sexual aggression (see Jones et al., 2020). Moreover, it highlights a key limitation of previous work in this area, namely, a failure to assess multiple psychological factors, including those that reliably predict sexual aggression amongst incarcerated persons (i.e., intimacy and social functioning deficits).

Guided by previous international research, we present two empirical studies that extend the knowledge base relevant to university-based sexual aggression and capture the nuances of sexual aggression amongst UK male university students. In Study 1, we establish through univariate analyses the multiple individual-level risk factors that differentiate sexually aggressive from non-sexually aggressive male students from one large plate glass university in the UK. We also examine using logistic regression modelling which factors most reliably predict students’ past sexual offending behaviors. Study 2 methodologically replicates Study 1 though uses a more diverse sample of male students from across the UK. This study allows us to externally validate our Study 1 findings, whilst also assessing the degree to which they generalize across the broader UK male student body. We extend past research by examining (a) the combined predictive value of psychological variables previously identified by international researchers as key indicators of university-based sexual aggression and (b) those variables not previously assessed with reference to university-based sexual aggression, but which have been shown to reliably predict sexual offending behaviors amongst incarcerated persons (i.e., intimacy and social functioning deficits).

It is worth noting that research has long demonstrated that university-based sexual aggression is multi-faceted and those who engage in it are often responding to various levels of influence on behavior (for a review, see Tharp et al., 2013; Dills et al., 2016). Given the gap in academic understanding regarding UK university students’ proabuse behaviors, in this paper, we made a purposeful decision to only assess attitudinal and personality-related indicators of sexual aggression. This allowed us to examine in-depth the psychological characteristics of perpetrators, which will help guide the development of more effective evidence-based harm prevention interventions. We will describe in future papers how more macro-level indicators (e.g., relationship, community, and societal-level factors) influence students’ proabuse behaviors, to further refine our understanding.

To encourage transparent and scientifically robust research practices, we pre-registered the hypotheses, research design, and data cleaning and analysis plans for both our studies via the Open Science Framework prior to data collection. These are publicly available via the following links, where you will also find copies of our materials, surveys, and raw data: https://osf.io/4ht8m/ (Study 1) and https://osf.io/n73wy/ (Study 2).

## Study 1

In Study 1, we assessed the psychological characteristics of sexually aggressive male students at a select university in South-East England. Based on previous research and theory, there were two hypotheses for this study. First, that the prevalence of sexual aggression would be higher amongst our sample compared to non-university males within the community (as reported in previous literature). Second, that there would be a difference in scores across psychological measures between male university students who had recently engaged in sexual aggression versus their non-offending peers. Specifically, we predicted that perpetrators would display greater aggression, alcohol consumption, hostility toward women, loneliness, rape myth acceptance, and sports involvement; lower assertiveness, emotion regulation, self-efficacy in romantic relationships, and self-esteem; and more atypical sexual fantasies. We further explored whether logistic regression modelling would highlight the risk factors that most reliably predict past sexual aggression amongst our sample and be able to discriminate at a greater-than-chance level between those who had and had not offended.

## Method

### Participants

Participants were adult students enrolled at a plate glass university in South-East England who identified as heterosexual males. They were recruited through opportunity sampling and reimbursed for their time with course credits or entered into a prize draw. In total, 259 students successfully completed our online survey entitled, “The Psychological and Behavioural Characteristics of University Males” (see our publicly available pre-registration for data cleaning exclusion criteria). Based on Peduzzi et al.’s (1996) established rule-of-thumb of 10 events per variable per outcome event, this sample size was sufficient for an adequately powered logistic regression model.

The age of participants ranged from 18 to 68 years (*M* = 22.9, *SD* = 6.6). The majority identified as White British (*n* = 151; 58.3%) and reported their highest educational attainment as A-Level or equivalent (*n* = 152; 58.7%). There were descriptive similarities between our sample and the university’s total male student body, as reported by centrally held university data. The only difference between groups was on highest educational attainment, *p* < .001 (see Supplementary Table S1 for post hoc pairwise comparisons).

### Overview of Measures

Measures comprised validated self-report instruments that assessed characteristics relevant to sexual aggression amongst either male university students in other countries or incarcerated males (see Fisher et al., 1999; Hanson & Bussière, 1998; Hanson & Morton-Bourgon, 2005; Mann et al., 2010; Marshall et al., 1997). These measures mapped onto key themes identified in the general sexual offending literature as being associated with sexual aggression, namely, inappropriate sexual interests, intimacy and social functioning deficits, offense-supportive cognitions, and self/emotional regulation issues.

We report in Table 1 the internal consistency (*α*) scores for continuous measures using George and Mallery’s (2016) classifications. Following a review of Clark and Watson (1995), it was decided that items that produced low (i.e., <.25) corrected item-total correlations across groups should be removed to increase scale reliability.^1^ This cut-off is less conservative than the one noted in our publicly available pre-registration (i.e., .30) and ensured that we avoided masking possible predictive factors.

**Table 1.** Internal Consistency and Mean Scores between SAs and NSAs across Studies 1 and 2 for Each Administered Measure.

Measure	Study 1			Study 2			Range^a^
	Cronbach’s α (SA, NSA)	SAs (*n* = 33)	NSAs (*n* = 226)	Cronbach’s α (SA, NSA)	SAs (*n* = 30)	NSAs (*n* = 265)	
		*M* (*SD*)	*M* (*SD*)		*M* (*SD*)	*M* (*SD*)	
Measure of sexual aggression							
SES-SFP	.82			.91			
Continuous measures							
BIDR-6-IM	.77 (.59, .77)	63.2 (12.6)	77.4 (14.6)	.77 (.76, .77)	70.4 (14.2)	73.4 (15.3)	20–140
BPAQ	.85 (.83, .83)	33.4 (9.5)	31.6 (9.7)	.86 (.77, .86)	37.4 (8.8)***	30.8 (9.7)	12–72
DERS-SF	.88 (.90, .88)	39.2 (11.5)	39.8 (11.1)	.91 (.80, .92)	37.8 (9.1)*	34.1 (11.8)	18–90
DJGL	.78 (.80, .78)	17.1 (5.0)	16.0 (4.7)	.79 (.70, .80)	16.7 (4.5)	15.9 (4.8)	6–30
HTW	.85 (.80, .85)	30.0 (7.6)**	25.7 (8.6)	.88 (.78, .88)	34.9 (8.3)***	26.2 (9.4)	10–70
IRMA-R	.89 (.88, .88)	44.1 (11.6)**	37.3 (10.0)	.90 (.88, .90)	46.0 (12.4)***	37.4 (11.1)	19–95
RSE_neg_	.83 (.83, .83)	12.8 (3.2)	13.0 (3.3)	.87 (.79, .88)	12.5 (3.0)	11.9 (3.5)	5–20
RSE_pos_	.86 (.88, .86)	10.5 (2.9)	10.1 (2.7)	.87 (.81, .87)	14.8 (2.7)	14.5 (2.8)	5–20
SERR^b^	.89 (.82, .89)	61.2 (13.6)	59.4 (16.3)	.90 (.87, .90)	56.2 (16.7)*	49.4 (18.2)	12–108
SFQ-R-SV	.82 (.82, .82)	10.3 (7.6)***	7.0 (6.1)	.87 (.90, .85)	12.9 (9.3)***	8.0 (6.8)	0–108
SRAS-SF^b^	.83 (.82, .84)	61.4 (13.2)	62.6 (14.2)	.83 (.75, .84)	65.7 (11.1)	64.0 (13.8)	19–114
Categorical measure		*Mdn*	*Mdn*		*Mdn*	*Mdn*	
AIM		2	2		2	2	1–4

*Note.* SA = sexual aggressor; NSA = non-sexual aggressor; SES-SFP = Sexual Experiences Survey–Short Form: Perpetration; IRMA-R = Illinois Rape Myth Acceptance Scale-Revised; SFQ-R-SV = Sexual Fantasy Questionnaire Revised–Short Version; DJGL = De Jong Gierveld Loneliness scales; HTW = Hostility Toward Women scale; RSE_neg_ = Rosenberg Self-Esteem scale (negative); RSE_pos_ = Rosenberg Self-Esteem scale (positive); BPAQ = Short-Form Buss-Perry Aggression Questionnaire; SERR = Self-Efficacy in Romantic Relationships scale; SRAS-SF = Simple Rathus Assertiveness Schedule–Short Form; DERS-SF = Difficulties in Emotion Regulation Scale–Short Form; BIDR-6-IM = Balanced Inventory of Desirable Responding-Version 6; AIM = Athletic Involvement Measure.

^a^Scale ranges are displayed in their original formats and have not been edited to reflect dropped items (see Footnote 1).

^b^These scales were recoded so that higher scores reflected nonconformity.

* *p* < .05 ** *p* < .01 *** *p* < .001.

### Measure of Sexual Aggression

*Sexual Experiences Survey–Short Form: Perpetration *(SES-SFP; Koss et al., 2007): A modified version of the SES-SFP assessed participants’ history of perpetrating sexually aggressive acts. Participants self-reported the number of times (0, 1, 2, or 3+) in the past 24 months they had engaged in 35 sexual outcome/tactic strings. This timeframe was chosen to ensure that we only captured acts that had occurred since the legal age of consent for sexual activity in the UK (currently 16 years), based on the lowest possible age of participants across our studies (i.e., 18 years). Each outcome/tactic string represented either an aberrant or illegal sexual behavior. An example outcome is “I had oral sex with someone or had someone perform oral sex on me without their consent by…” and an example tactic is “…threatening to physically harm them or someone close to them.” Based on their responses, participants were classed into up to four mutually exclusive categories of sexual perpetration: “none,” “unwanted sexual contact,” “sexual coercion,” and “rape/attempted rape.” A follow-up item asked self-reported sexually aggressive participants the sex of their victim(s).

As suggested by Anderson et al. (2017), a dichotomous scoring agenda was used to measure SES-SFP responses. Participants who emphatically rejected survey items were classed as “non-sexual aggressors” (NSAs), whilst those who provided any non-zero response were classed as “sexual aggressors” (SAs). The SES-SFP has demonstrated excellent internal consistency with university males in the US (e.g., Johnson et al., 2017), as well as structural and convergent validity (Anderson et al., 2017).

### Inappropriate Sexual Interests

*Sexual Fantasy Questionnaire Revised*–*Short Version *(SFQ-R-SV; Bartels & Harper, 2018): Atypical sexual fantasies were assessed using a modified version of the SFQ-R-SV, comprising items from the “Masochistic,” “Sadistic,” “Impersonal,” and “Pre/Tactile Courtship Disorder” clusters (27 items in total). The “Romantic” and “Bodily Functions” clusters were not included as they are not regularly endorsed by community samples (Bartels & Harper, 2018). Using a 5-point Likert scale from *Have never fantasized about* (0) to *Have fantasized about very frequently* (4), participants reported how often they had fantasized about each of the sexual behaviors described. Scores were analyzed collectively and can range from 0 to 108, with higher scores indicating a greater endorsement of items. Example items include “Being physically hurt” (Masochistic cluster) and “Torturing others” (Sadistic cluster).

### Intimacy and Social Functioning Deficits

*De Jong Gierveld Loneliness Scale *(DJGL; De Jong Gierveld & Van Tilburg, 2006): This six-item scale was used to assess overall loneliness. Participants responded to items using a psychometrically validated response format anchored by *No!* (1) and *Yes!* (5). Scores can range from 6 to 30, with higher scores indicating greater perceived loneliness. An example item is “I often feel rejected.”

*Rosenberg Self-Esteem Scale *(RSE; Rosenberg, 1979): This 10-item measure assessed the construct of global self-esteem. Participants responded to items using a 4-point Likert scale from *Strongly agree* (1) to *Strongly disagree* (4). Scores can range from 10 to 40, with higher scores indicating greater self-esteem. Our psychometric analyses suggested that the scale comprised two factors: negative self-esteem (which mapped onto reverse-coded items) and positive self-esteem. Therefore, the RSE was split into the RSE_neg_ and RSE_pos_ and treated as two distinct scales during analyses. Example items include “I certainly feel useless at times” (RSE_neg_ scale) and “On the whole, I am satisfied with myself” (RSE_pos_ scale).

*Self-Efficacy in Romantic Relationships Scale *(SERR; Riggio et al., 2013): This 12-item measure assessed general feelings of relationship self-efficacy, independent of actual romantic relationships or intimate partnerships. Participants responded to items on a 9-point Likert scale from *Strongly disagree* (1) to *Strongly agree* (9). Scores can range from 12 to 108, with higher scores indicating greater self-efficacy in romantic relationships. An example item is “Romantic relationships are very difficult for me to deal with.”

*Simple Rathus Assertiveness Schedule–Short Form *(SRAS-SF; Jenerette & Dixon, 2010): This 19-item measure assessed levels of assertiveness. Participants responded to items on a 6-point Likert scale from *Very much unlike me* (1) to *Very much like me* (6). Scores can range from 19 to 114, with higher scores indicating greater assertiveness. An example item is “Most people stand up for themselves more than I do.”

### Offense-supportive Cognitions

*Hostility Toward Women Scale *(HTW; Lonsway & Fitzgerald, 1995): This 10-item scale assessed general hostility toward women. Participants responded to items using a 7-point Likert scale anchored by *Strongly disagree* (1) and *Strongly agree* (7). Scores can range from 10 to 70, with higher scores indicating greater hostility. An example item is “I think that most women would lie just to get ahead.”

*Illinois Rape Myth Acceptance Scale–Revised *(IRMA-R; McMahon & Farmer, 2011): This 19-item measure is designed to assess subtle rape myths. Participants responded to each item on a 5-point Likert scale from *Strongly agree* (1) to *Strongly disagree* (5). Scores can range from 19 to 95, with higher scores indicating greater rape myth acceptance. An example item is “If a girl doesn’t say ‘no’ she can’t claim rape.”

### Self/Emotional Regulation Issues

*Short-Form Buss-Perry Aggression Questionnaire *(BPAQ; Bryant & Smith, 2001): This 12-item measure assessed general aggression. Participants responded to items on a 6-point Likert scale from *Extremely uncharacteristic of me* (1) to *Extremely characteristic of me* (6). Scores can range from 12 to 72, with higher scores indicating greater aggression. An example item is “Sometimes I fly off the handle for no good reason.”

*Dailing Drinking Questionnaire *(DDQ): This is an adapted version of Collins et al.’s (1985) established measure that assesses individual alcohol consumption behaviours. Drinks are split into 10 categories based on their units. Responses across categories are summed so that researchers can assess the average volume, quantity, and frequency of alcohol consumed by an individual over any given period. In our study, the DDQ was used to probe average daily alcohol intake over the past 3 months.

*Difficulties in Emotion Regulation Scale*–*Short Form *(DERS-SF; Kaufman et al., 2016): This 18-item measure assessed emotion regulation deficits. Participants responded to items using a 5-point Likert scale from *Almost never* (1) to *Almost always* (5). Scores can range from 18 to 90, with higher scores indicating greater deficits in emotion regulation. An example item is “When I’m upset, I have difficulty controlling my behaviour.”

## Additional Measures

*Athletic Involvement Measure *(AIM): We used a modified version of Koss and Gaines’ (1993) recognized measure to assess participants’ level of sports participation. This asked respondents which of four descriptions best suited their current participation level: “I do not participate in any sports,” “I only participate in sports informally (i.e., I play sports, but I am not a member of a sports club or sports society),” “I am a member of and play for one sports club or sports society,” or “I am a member of and play for more than one sports club or sports society.” Each item accrues one mark; therefore, scores can range from 0 to 3 with higher scores indicate greater sports involvement.

*Balanced Inventory of Desirable Responding–Version 6 *(BIDR-6-IM; Paulhus, 1988): We used the “Impression Management” scale from Paulhus’ (1988) BIDR-6 to assess participants’ tendency to inflate positively their self-image—an indicator of possible biased responding to the SES-SFP. Participants responded to 20 items using a 7-point Likert scale from *Strongly disagree* (1) to *Strongly agree* (7). Scores can range from 20 to 140, with higher scores indicating a greater tendency toward impression management. An example item is “I always obey laws, even if I’m unlikely to get caught.”

### Procedure

Ethical approval was granted by our university (Ref: 201815460056315287). Participants accessed our survey through Qualtrics in their own time. A screening measure initially assessed study eligibility. Participants then read an information sheet and responded to a consent form, a demographic survey (which collected non-identifiable personal data), and our battery of measures. Four attention check items were included to assess individual concentration and participants were required to respond to each measure in full. After completing the study, participants were debriefed and informed of a UK website dedicated to supporting past, current, and potential sexual aggressors (https://www.stopitnow.org.uk).

### Analysis Plan

Analyses were conducted on SPSS 24 for Windows. To aid interpretation of results, the SERR and SRAS-SF were recoded so higher scores reflected nonconformity. Data that were not normally distributed or displayed non-monotonic relationships (i.e., the DERS-SF, HTW, and SFQ-R-SV) were transformed, as recommended by Tabachnick and Fidell (2013). Subsequently, we present in our results the ratio of the difference in mean scores for SAs and NSAs on these measures, versus actual mean scores. We used Van Selst and Jolicoeur’s (1994) *z*-score criterion for univariate outlier detection; of 20 possible outliers, three were retained (unadjusted), five were excluded, and 12 were winsorized, which reduced distributional problems within our dataset. Unfortunately, several participants self-reported their cumulative, not average, daily alcohol intake over the past 3 months on the DDQ. As we could not differentiate between those who did and did not respond correctly, we had to exclude this measure from our analyses

## Results

### Sexual Aggression: Prevalence and Features

In total, 33 participants (12.7% of the sample) self-reported having perpetrated 106 sexually aggressive acts over the past 24 months. Sexual coercion comprised the largest category of reported act (41.5% of all reported acts), having been perpetrated by 14 participants (6.2% of the sample). This was followed by unwanted sexual contact (34.9%) and rape/attempted rape (23.6%), which were perpetrated by 10.2% (*n* = 23) and 6.2% (*n* = 14) of the sample, respectively. Most SAs (*n* = 13; 39.4%) committed two sexually aggressive acts in total, although a considerable number (*n* = 11; 33.3%) reported three or more. A majority of SAs (*n* = 27; 81.8%) self-reported female victims only, though five (15.2%) reported both female and male victims, and one SA (3.0%) reported a male victim.

It is worth noting that participants who responded “3+” to any of the outcome/tactic strings on the SES-SFP were recorded as having committed only three sexually aggressive acts. Therefore, the above figures likely represent conservative estimates of prevalence, as some participants who responded “3+” may have committed more than three sexual acts.

### Group Comparisons

The survey responses of SAs and NSAs were compared to assess which psychological variables should enter our logistic regression model. We also evaluated group differences on demographic variables, based on their established link with sexual aggression perpetration amongst incarcerated persons (see Hanson & Bussière, 1998). Multiple test corrections were not applied to avoid masking possible predictive factors.

*Demographic Variables*: Ostensibly, there were demographic similarities between SAs and NSAs, and univariate analyses showed that participants could only be differentiated by their ethnicity, *p* = .048 (see Supplementary Table S2 for post hoc pairwise comparisons). Given recent contentions that ethnicity may explain sexual aggression through social or cultural norms (see Palmer et al., 2020), we decided to include this variable in our logistic regression model.

*Psychological Measures*: Descriptive statistics were computed separately for SAs and NSAs (see Table 1). Welch’s t-tests showed that both groups could only be differentiated by their scores on the HTW (*M*_*ratio*_ = 0.2, 95% CI [0.03 to 0.51], *t*(46.52) = 3.18, *p* = .003, *d* = 0.51), SFQ-R-SV (*M*_*ratio*_ = 0.6, 95% CI [0.30 to 1.05], *t*(56.57) = 4.30, *p* < .001, *d* = 0.52), and IRMA-R (*M* = 6.8, 95% CI [2.48 to 11.06], *t*(39.31) = 3.19, *p* = .003, *d* = 0.66). A chi-square test of homogeneity could not differentiate between groups on the AIM.

*Impression Management*: Results showed that there was no significant relationship between BIDR-6-IM scores and scores on the SES-SFP for SAs.

*Classifying Sexual Aggressors*: To assess their ability to predict sexual aggression, the variables that differentiated between SAs and NSAs were force entered into a binomial logistic regression model. As it contained multiple cell counts less than five, ethnicity was dichotomized into a “White British” and a “minority ethnicity” category. Assumption testing highlighted nine SAs as multivariate outliers for having standardized residuals greater than ±3 standard deviations—after inspection, five were omitted. Youden’s Index (*J*) was calculated to derive an optimal cut-off for model construction, which suggested a value of .088.

The model was significant, *χ*^2^(4) = 25.82, *p* < .001, and explained between 9.7% (Cox & Snell R^2^) and 19.3% (Nagelkerke R^2^) of variance in sexual aggression. Overall, 65.0% of all cases were correctly classified. Of the predictor variables, only the IRMA-R and SFQ-R-SV made a significant contribution (see Table 2). Receiver operating characteristic (ROC) curve analysis revealed that the model could discriminate between SAs and NSAs at better-than-chance level; area under the curve (AUC) = .77, *p* < .001, 95% CI [.68, .85], corresponding to a large Cohen’s *d* effect size of approximately 1.04 (Rice & Harris, 2005).^2^

**Table 2.** Final Logistic Regression Models for Studies 1 and 2 Predicting the Likelihood of Self-reported Sexual Aggression.

Measure	β	*SE*	*Wald*	*df*	*p*	*OR*	95% CI for OR	
							*LL*	*UL*
Study 1								
HTW	0.01	0.03	0.06	1	.81	1.01	0.95	1.07
IRMA-R	0.08	0.03	8.48	1	.004	1.08	1.03	1.14
SFQ-R-SV	0.07	0.03	6.07	1	.01	1.08	1.02	1.14
Ethnicity	0.27	0.44	0.36	1	.55	1.31	0.55	3.10
Constant	−6.32	1.07	34.73	1	<.001	0.00		
HL goodness of fit: χ^2^(8) = 2.54, *p* = .96								
Study 2								
BPAQ	0.11	0.04	10.33	1	.001	1.12	1.05	1.20
HTW	0.14	0.03	18.51	1	<.001	1.15	1.08	1.22
SFQ-R-SV	0.12	0.03	13.33	1	<.001	1.12	1.06	1.20
Constant	−12.51	2.11	35.09	1	<.001	0.00		
HL goodness of fit: χ^2^(8) = 4.81, *p* = .78								

*Note. OR* = odds ratio; CI = confidence interval; *LL* = lower limit; *UL* = upper limit; IRMA-R = Illinois Rape Myth Acceptance Scale-Revised; SFQ-R-SV = Sexual Fantasy Questionnaire Revised–Short Version; HTW = Hostility Toward Women scale; BPAQ = Short-Form Buss-Perry Aggression Questionnaire.

## Study 2

Study 2 was pre-registered as a replication of Study 1 with minor modifications. Most notably, we used a broader independent sample of male students from across UK universities to assess sexual aggression, which allowed us to examine the psychological characteristics of SAs nationally, as well as the generalizability of our Study 1 findings. As a result of our new recruitment method, we also modified our methodology in the ways described below to increase the validity of our data. Our hypotheses remained unchanged from Study 1.

## Method

### Participants

Participants were recruited on the crowdsourcing platform Prolific (see Palan & Schitter, 2018), which allowed access to a large pool of eligible participants. We chose Prolific as recent evaluations have shown that users of the site generate high-quality and accurate data and are often more naïve than participants on other platforms (Peer et al., 2017, 2021). Prolific also overcomes the drawbacks of more traditional data collection methods when it comes to assessing stigmatizing sexual behaviors (Ó Ciardha et al., 2021).

Relevant pre-screening filters were set on Prolific to capture participants who were adult university students residing in the UK and who identified as heterosexual males (*N* = 688). To maximize the constraint of our final model’s parameters, and to ensure a sufficient sample of SAs for Study 3, we purposively recruited more participants here than in Study 1. Therefore, our final sample comprised *n* = 295 students (42.9% of the eligible target population on Prolific; see our pre-registration for data cleaning exclusion criteria).

The age of participants ranged from 18 to 75 years (*M* = 25.1, *SD* = 8.3). As in Study 1, the majority identified as White British (*n* = 208; 70.5%) and reported their highest level of educational achievement as A-Level or equivalent (*n* = 135; 45.8%). Overall, students from 100 (out of 161) UK universities participated. There were descriptive similarities between our sample and the UK male university student body, as reported by the Higher Education Student Statistics: UK, 2017/18 survey (Higher Education Statistics Agency, 2019). The only group differences were on highest educational attainment, *p* = .004, and university country, *p* = .008 (see Supplementary Table S3 for post hoc pairwise comparisons).

### Measures and Procedure

Study 2 was ethically approved as previously (Ref: 201915651873045842). Participants completed the survey as in Study 1. Two new items were included: one in the demographic survey that asked for university affiliation, and one at the end of the SES-SFP that asked SAs for their relationship to their victim(s). Based on our Study 1 findings, the completion time for the survey was set at 25-minutes and the maximum allowed time as 60-minutes. Participants received compensation at a pro-rated rate of £5.00 per hour. Demographic survey data were used to corroborate participants’ responses to the pre-screening filters. As shown in Table 1, internal consistency scores across measures were markedly better than in Study 1.

### Analysis Plan

Using the methods described in Study 1, 18 possible univariate outliers were identified; three were retained (unadjusted), one was excluded, and 14 were winsorized, which resulted in positive statistical gains. The HTW and SFQ-R-SV were transformed; subsequently, we present in our results the ratio of the difference in mean scores for SAs and NSAs on these measures, versus actual mean scores.

## Results

### Sexual Aggression: Prevalence and Features

In total, 30 participants (10.1% of the sample) self-reported having perpetrated 145 sexually aggressive acts over the past 24 months (though, as noted earlier, this could be a conservative estimate). As in Study 1, sexual coercion comprised the largest category of reported act (37.9% of all reported acts), having been perpetrated by 18 participants (6.1% of the sample). This was followed by rape/attempted rape (35.9%; notably higher than Study 1) and unwanted sexual contact (26.2%), which were perpetrated by 5.4% (*n* = 16) and 4.7% (*n* = 14) of the sample, respectively. Unlike Study 1, most SAs (*n* = 12; 40.0%) reported three or more sexually aggressive acts. As previously, most acts were committed against females only (*n* = 26; 86.7%), though four SAs (13.3%) reported both female and male victims. Victims were mainly other students (80.0% of cases) known to the participant (66.7% of cases).

### Group Comparisons

*Demographic Variables*: Again, there were demographic similarities between SAs and NSAs in this study (see Supplementary Table S2). As in Study 1, our sample displayed a preponderance toward younger, highly educated students who identified as White British. Unlike earlier, univariate analyses showed that SAs and NSAs could not be differentiated on any of the four demographic variables.

*Psychological Measures*: Again, descriptive statistics were computed separately for both groups and showed that, across measures, SAs consistently scored higher than NSAs (see Table 1). As in Study 1, univariate analyses showed that both groups could be differentiated by their scores on the HTW (*M*_*ratio*_ = 0.7, 95% CI [0.30 to 1.26], *t*(40.37) = 5.83, *p* < .001, *d* = 0.94), SFQ-R-SV (*M*_*ratio*_ = 0.8, 95% CI [0.35 to 1.30], *t*(42.43) = 4.30, *p* < .001, *d* = 0.70), and IRMA-R (*M* = 8.5, 95% CI [3.73 to 13.34], *t*(34.46) = 3.61, *p* < .001, *d* = 0.76). Unlike Study 1, differences in scores were also highlighted on the BPAQ (*M* = 6.6, 95% CI [3.14 to 10.11], *t*(37.44) = 3.85, *p* < .001, *d* = 0.69), SERR (*M* = 6.8, 95% CI [0.24 to 13.42], *t*(37.26) = 2.10, *p* = .04, *d* = 0.38), and DERS-SF (*M* = 3.8, 95% CI [0.12 to 7.46], *t*(40.74) = 2.09, *p* = .04, *d* = 0.33). No significant differences between groups were found on the remaining psychological measures, including the AIM.

*Impression Management*: As in Study 1, univariate testing highlighted that there was no significant relationship between BIDR-6-IM and SES-SFP scores for SAs.

*Classifying Sexual Aggressors*: Owing to a low *n* in the SA group (which would reduce the power of our logistic regression analyses), a hierarchical logistic regression model was initially run to assess which of the six significant variables from our univariate tests could predict sexual aggression and should be carried forward to our main analysis. Variables were entered individually in blocks based on their *p*-values. This hierarchical model highlighted that IRMA-R, SERR, and DERS-SF scores did not significantly improve the model’s fit and therefore should be excluded. To assess their ability to predict sexual aggression, the remaining variables were force entered into a binomial logistic regression model, as in Study 1. Assumption testing highlighted seven SAs as multivariate outliers, which were omitted from the analyses. Here, a classification cut-off value of *J* = .113 was used.

The final logistic regression model was significant, *χ*^2^(3) = 57.63, *p* < .001, and explained between 18.1% (Cox & Snell R^2^) and 42.5% (Nagelkerke R^2^) of variance in sexual aggression. Overall, 85.1% of cases were correctly classified. Unlike in Study 1, all predictor variables made a significant contribution (see Table 2). ROC curve analysis revealed that the model could discriminate between groups at better-than-chance level; AUC = .93, *p* < .001, 95% CI [.89, .96], corresponding to a large Cohen’s *d* effect size of approximately 2.09 (Rice & Harris, 2005).^3^

### Discussion

Our studies represent the first empirical assessment of the risk factors associated with university-based sexual aggression in the UK and offer the first reported estimate of the prevalence of sexual aggression perpetrated by UK male university students. They extend past US research by examining the combined influence of both new and established psychological variables on male students’ proabuse behaviors, including those associated with sexual abuse perpetration amongst incarcerated persons. Taken together, our findings highlight that male university students in the UK with a history of sexual aggression comprise a distinct forensic population, who can be differentiated from their non-offending peers by various psychological indicators associated with their past proabuse behaviors.

Across Studies 1 and 2, 11.4% (*n* = 63) of our combined sample (*n* = 554) self-reported having committed at least one sexually aggressive act in the past 24 months, for a total of 251 illegal sexual acts overall. These findings mirror those reported in large US studies into campus sexual assault, where between 11.5%-17.9% of male university students disclose having engaged in sexually aggressive behaviors recently (Abbey & McAuslan, 2004; Gidycz et al., 2007; Mouilso & Calhoun, 2016). They are also comparable to estimates of prevalence from research conducted with male students in other European countries, including Germany (13.3% prevalence; Krahé & Berger, 2013), Poland (11.7% prevalence; Tomaszewska & Krahé, 2018), and Spain (15.3% prevalence; Martín et al., 2005). No analogous research has been conducted in the UK; however, the prevalence of self-reported sexual aggression is notably higher amongst our participants compared to non-university males in the community, where 7.3.% disclose a history of such behaviors (Krahé et al., 2014). This supports prior contentions (e.g., Bloom et al., 2021) that universities are a breeding ground for sexual aggression and emphasizes the critical need for better harm prevention initiatives on campuses, including more evidence-based psychological interventions for male students who are at risk of offending.

Findings also support our hypotheses that there would be differences in scores across psychological measures between SAs and NSAs. While descriptive comparisons of mean scores between groups support this prediction, inferential analyses differentiated between individuals who had and who had not recently perpetrated sexual aggression on select variables only; specifically, measures of hostility toward women, atypical sexual fantasies, rape myth acceptance, and ethnicity (Study 1), and hostility toward women, atypical sexual fantasies, rape myth acceptance, aggression, self-efficacy in romantic relationships, and difficulties in emotion regulation (Study 2). When entered into a logistic regression model, only atypical sexual fantasies and rape myth acceptance (Study 1), and hostility toward women, atypical sexual fantasies, and aggression (Study 2) predicted sexual aggression. In support of our hypotheses, both models could discriminate between SAs and NSAs at greater-than-chance level; however, the model in Study 2 correctly classified more cases.

Our findings support campus sexual assault studies from other countries, which have highlighted key psychological differences between males who have and have not engaged in recent sexual aggression in terms of specific attitudinal, personality, and experiential risk-related factors (e.g., Abbey et al., 2001; Čvek & Junaković, 2020; D’Abreu & Krahé, 2014; Thompson et al., 2013). Given arguments that male sexual aggression is driven by hypermasculinity and adversarial sexual beliefs (see Abbey & McAuslan, 2004; Chan, 2021; Čvek & Junaković, 2020; Martín et al., 2005), it is unsurprising that high levels of hostility toward women, rape myth acceptance, and atypical sexual fantasies predicted past engagement in the behavior in our sample. To this end, our findings support the confluence model (Malamuth et al., 2021), which proposes that hostile masculinity—a pronounced obedience to traditional gender role beliefs for men—forms one of two key pathways to sexual aggression. Literature has also shown that increased aggression in males is a precursor to sexually aggressive expressions of behavior (see Rapaport & Burkhart, 1984), thus accounting for the ability of BPAQ scores to predict sexual aggression in Study 2.

### Implications for Sexual Harm Prevention Work on Campuses

Our logistic regression analyses show that sexually aggressive male university students in the UK are likely to be motivated by their high levels of rape myth acceptance, hostility toward women, and aggression. Harm prevention initiatives for this group should therefore target their negative and derogatory beliefs and encourage them toward more prosocial cognitions (e.g., by promoting positive regard for women and dispelling pervasive rape myths). Modules in empathy, and which contain a norms correction component, could also be beneficial in helping participants to understand their victim’s feelings and promote more prosocial thoughts. Whilst their value in sexual offender treatment programs is subject to debate (see Mann & Barnett, 2013), such modules have demonstrated success in reducing sexual aggression amongst students in previous empirical work (e.g., Gidycz et al., 2011).

As in past international research (e.g., Chan, 2021; Greendlinger & Byrne, 1987), atypical sexual fantasies associated with harmful or coercive sexual behaviors also predicted sexual aggression amongst our sample. Psychological interventions using covert sensitization and satiation demonstrate success in modifying atypical sexual fantasies among incarcerated persons (see Bartels & Gannon, 2011); however, these approaches are likely to be difficult to implement in university settings. More general arousal modification techniques may offer one solution (see Prentky & Knight, 1991), though research needs to be conducted first to assess their utility with student samples.

For maximum efficacy, it would be wise to embed any psychological interventions for students who have committed—or show a proclivity toward—sexual aggression within validated pre-existing harm prevention programs for university students (e.g., bystander or gender-transformative initiatives; see Bonar et al., 2020). In the UK, several universities have developed sexual violence reduction programs (see Universities UK, 2019); however, these often lack standardization, are not empirically informed, or derive from research with US male university students. Whole-university sexual violence campaigns have shown greater promise—particularly at reducing rape myth acceptance and increasing awareness of sexual violence amongst students—and may also offer promising avenues for reducing rates of university-based sexual aggression (e.g., Thomson et al., 2020). Our findings will help inform the development of more robust and evidence-based UK-derived initiatives.

### Limitations and Future Directions

Taken together, our studies offer a preliminary insight into the prevalence of, and psychological risk factors associated with, sexual aggression amongst male university students in the UK. Whilst our findings have exciting implications for the design of effective evidence-based harm prevention initiatives, we urge readers to consider them in the context of our studies’ limitations, which we outline below.

First, we assessed only “individual-level” risk factors (i.e., attitudinal and personality-related indicators; see Dills et al., 2016) associated with participants’ proabuse behaviors. This was a purposeful decision based on the lack of academic research into sexual aggression on UK campuses (see Jones et al., 2020) and our desire to examine in-depth the personal characteristics of SAs. However, it is well-established that university-based sexual aggression is multi-faceted in nature and often the result of many levels of influence on behavior (e.g., Dills et al., 2016; Tharp et al., 2013). To this end, it would be sensible for future researchers to examine how known relationship, community, and societal-level risk factors affect UK male students’ proabuse behaviors. Understanding more about the complex interplay between these factors will guide campus sexual harm prevention work, as well as the development of more effective interventions for students at risk of perpetration.

Second, our data were cross-sectional and assessed the psychological characteristics of SAs at one time point only. This meant we precluded assumptions about temporal sequencing and the possibility that risk factors interact in an ordered fashion during sexual aggression perpetration. Research examining male-perpetrated campus sexual assault in the US has demonstrated that there are time-varying risk factors associated with sexual aggression (Thompson et al., 2015); therefore, it would be expedient for researchers to conduct longitudinal investigations with male students in the UK.

Third, while we met minimum sample size recommendations for our inferential tests and logistic regression models (see Peduzzi et al., 1996), some analyses could have benefited from additional power. Low power was a result of there being more NSAs than SAs within our sample (a common complaint in sexual aggression research; see Swartout et al., 2011). We encourage future researchers to consider this limitation when designing study protocols, to ensure the validity of their findings. Future research studies adopting broader samples would further allow us to assess the generalizability of our results, which may be impacted as a result of our discrepancy in group sizes and low *N* overall.

Fourth, our studies—like most sexual aggression research—focused on males as perpetrators and females as victims. Other arrangements are of course possible. This is highlighted by Studies 1 and 2, where five and four participants, respectively, self-reported perpetrating sexual aggression against male victims also. To this end, we support focused follow-up research with individuals who have perpetrated university-based sexual aggression against male students and non-binary students (either wholly or in part), to ensure the development of effective and inclusive campus-wide harm prevention initiatives. This is critically important given that research has shown that sexual violence disproportionately affects members of marginsalized communities (e.g., Ellis, 2009; Revolt Sexual Assault, 2018).

Last, the predictors of sexual aggression differed between Studies 1 and 2, suggesting possible disparities in the psychological characteristics of SAs at different universities. This would have an obvious implication for harm prevention provisions across universities for male students who have displayed harmful sexual behaviors and suggests that a “one-size-fits-all” approach to intervention may not be effective. Replication studies adopting a broader sample would be valuable for confirming this finding and providing more robust assessments of the key psychological predictors associated with sexual aggression amongst male university students in the UK. To this end, future researchers may find it sensible to employ a range of data collection methods to ensure they recruit a representative sample of participants (e.g., those from minority groups or without access to online crowdsourcing platforms).

## Supplemental Material

sj-pdf-1-sax-10.1177_10790632211051682 – Supplemental Material for Understanding Sexual Aggression in UK Male University Students: An Empirical Assessment of Prevalence and Psychological Risk Factors Click here for additional data file. Supplemental Material, sj-pdf-1-sax-10.1177_10790632211051682 for Understanding Sexual Aggression in UK Male University Students: An Empirical Assessment of Prevalence and Psychological Risk Factors by Samuel T. Hales and Theresa A. Gannon in Sexual Abuse: A Journal of Research and Treatment

## Notes

1 From Study 1, items 3, 5, and 15 on the SFQ-R-SV, items 3 and 5 on the SERR, item 5 on the SRAS-SF, item 13 on the DERS-SF, and item 14 on the BIDR-6-IM were excluded. From Study 2, item 6 on the SFQ-R-SV, item 5 on the SRAS-SF, items 13, 14, and 15 on the DERS-SF, and item 10 on the BIDR-6-IM were excluded.

2 A model excluding participants who failed attention check items (*n* = 29) and which contained IRMA-R, SFQ-R-SV, and HTW scores was also significant, *χ*^2^(4) = 16.40, *p* = .003, and highlighted IRMA-R and SFQ-R-SV scores as significant predictor variables (*p* = .03 and *p* = .03, respectively). However, this model had a worse fit, *χ*^2^(8) = 7.13, *p* = .52, than our full model.

3 A model excluding participants who failed attention check items (*n* = 14) and which similarly contained HTW, SFQ-R-SV, and BPAQ scores was also significant, χ^2^(3) = 51.56, *p* < .001, and highlighted all three measures as significant predictor variables (*p* < .001, *p* < .001, and *p* = .005, respectively). Whilst the model had a marginally better fit, *χ*^2^(8) = 4.55, *p* = .80, than our full model, it explained less variation in sexual aggression scores (17.2% [Cox & Snell R^2^] and 41.1% [Nagelkerke R^2^]).
