# Supplementary material for: Understanding Sexual Aggression in UK Male University Students: An Empirical Assessment of Prevalence and Psychological Risk Factors
Source: Sex Abuse. 2021 Oct 27;34(6):744–70. doi: 10.1177/10790632211051682 (PMC9379390; doi:10.1177/10790632211051682)
Supplement: sj-pdf-1-sax-10.1177_10790632211051682 – Supplemental Material for Understanding Sexual Aggression in UK Male University Students: An Empirical Assessment of Prevalence and Psychological Risk Factors [file sj-pdf-1-sax-10.1177_10790632211051682.pdf]

Supplementary Table S1

Demographic Comparisons between our Study 1 Sample and the Male Student Body at the Selected University, as Reported by Centrally Held University Data

Variable	Study sample (*N* = 259)		University male student body[a] (*N* = 9,100)	
	n	%	*n*	%
Age				
20 and under	112	43.24	3,700	40.66
21-30	125	48.26	4,725	51.92
31-40	13	5.02	405	4.45
41-50	5	1.93	180	1.98
51-60	3	1.16	60	0.66
61-70	1	0.39	30	0.33
Ethnicity[b c]				
White	189	72.98	5,520	62.48
Black African	14	5.41	910	10.30
Black Caribbean	3	1.16	150	1.70
Mixed White/Asian	6	2.32	170	1.92
Mixed White/Black African	1	0.39	60	0.68
Mixed White/Black Caribbean	2	0.77	85	0.96
Mixed - Other	6	2.32	195	2.21
Arab	3	1.16	100	1.13
Bangladeshi	3	1.16	95	1.08
Chinese	6	2.32	415	4.70
Indian	11	4.25	420	4.75
Pakistani	2	0.77	135	1.53
Asian - Other	11	4.25	405	4.58
Any - Other	2	0.77	175	1.98
Highest educational attainment[b c]				
GCSE or equivalent	4	1.54	75	0.91
A-Level or equivalent	152***	58.69	6,855	82.79
B.A. or equivalent	55***	21.24	715	8.64
Postgraduate degree or equivalent	44***	16.99	510	6.16
Other	4	1.54	125	1.51

Note. Figures may not add up to 100% due to rounding.
[a] Data rounded to the nearest five students. [b] Due to missing data and "Prefer not to answer" responses, totals in the *University* column may not always equal 9,100. [c] Due to University data collection methods, some categories have been collapsed.
***$p < .001$

Supplementary Table S2

Demographic Comparisons between SAs and NSAs in Studies 1 and 2

Variable	Study 1		Study 2	
	SA (*n* = 33)	NSA (*n* = 226)	SA (*n* = 30)	NSA (*n* = 265)
	n (%)	*n* (%)	*n* (%)	*n* (%)
Age				
20 and under	13 (39.39)	99 (43.81)	7 (23.33)	85 (32.08)
21-30	18 (54.55)	107 (47.35)	17 (56.67)	135 (50.94)
31-40	2 (6.06)	11 (4.87)	6 (20.00)	30 (11.32)
41-50	0	5 (2.21)	0	8 (3.02)
51-60	0	3 (1.33)	0	4 (1.51)
61+	0	1 (0.44)	0	3 (1.13)
Ethnicity				
White British	16 (48.48)	135 (59.73)	20 (66.67)	188 (70.94)
White Irish	1 (3.03)	5 (2.21)	0	1 (0.38)
White - Other	3 (9.09)	29 (12.83)	3 (10.00)	21 (7.92)
Black African	1 (3.03)	13 (5.75)	2 (6.67)	9 (3.40)
Black Caribbean	1 (3.03)	2 (0.88)	0	2 (0.75)
White/Asian	0	6 (2.65)	0	1 (0.38)
White/Black African	0	1 (0.44)	1 (3.33)	4 (1.51)
White/Black Caribbean	0	2 (0.88)	0	1 (0.38)
Mixed - Other	1 (3.03)	5 (2.21)	0	3 (1.13)
Arab	1 (3.03)	2 (0.88)	1 (3.33)	0
Bangladeshi	0	3 (1.33)	1 (3.33)	3 (1.13)
Chinese	0	6 (2.65)	0	8 (3.02)
Indian	2 (6.06)	9 (3.98)	0	9 (3.40)
Pakistani	1 (3.03)	1 (0.44)	1 (3.33)	4 (1.51)
Asian - Other	6 (18.18)***	5 (2.21)	1 (3.33)	7 (2.64)
Any - Other	0	2 (0.88)	0	1 (0.38)
Highest educational attainment				
GCSE or equivalent	0	4 (1.77)	1 (3.33)	8 (3.02)
A-Level or equivalent	20 (60.61)	132 (58.41)	13 (43.33)	122 (46.04)
B.A. or equivalent	9 (27.27)	46 (20.35)	10 (33.33)	87 (32.83)
M.A. or equivalent	2 (6.06)	37 (16.37)	5 (16.67)	40 (15.09)
Ph.D. or equivalent	1 (3.03)	4 (1.77)	1 (3.33)	5 (1.89)
Other	1 (3.03)	3 (1.33)	0	1 (0.38)
University country				
England			21 (70.00)	194 (73.21)
Scotland			4 (13.33)	21 (7.92)
Wales			2 (6.67)	17 (6.42)
Northern Ireland			0	2 (0.75)
Open University			1 (3.33)	23 (8.68)

Note. Figures may not add up to 100% due to rounding or participants providing "Prefer not to answer" responses. SA = Sexual aggressor; NSA = Non-sexual aggressor.
***$p < .001$

Supplementary Table S3

Demographic Comparisons between our Study 2 Sample and the UK University Male Student Body, as Reported by the Higher Education Student Statistics: UK, 2017/18 survey (Higher Education Statistics Agency, 2019)

Variable	Study sample (*N* = 295)		UK male student body (*N* = 1,007,730)	
	n	%	*n*	%
Age [a]				
20 and under	92	31.19	415,923	41.27
21-24	103	34.92	281,719	27.96
25-29	41	13.90	111,513	11.07
30+	59	20.00	198,482	19.70
(Did not respond)	(0)	(0)	(97)	(0.01)
Ethnicity [a b]				
White	233	78.98	609,802	75.23
Black	14	4.75	57,455	7.09
Asian	35	11.86	86,697	10.70
Mixed	9	3.05	31,110	3.84
Other	1	0.34	12,683	1.56
(Did not respond)	(3)	(1.02)	(12,782)	(1.58)
Highest educational attainment [a c]				
No formal qualification	0	0	5,785	1.73
GCSE or equivalent and below	9	3.05	7,423	2.23
A-Level or equivalent	135	45.76	178,346	53.46
B.A. or equivalent	97	32.88	109,205	32.74
Postgraduate degree or equivalent	51**	17.29	27,098	8.12
Other	1	0.34	2,770	0.83
(Did not respond)	(2)	(0.68)	(2,972)	(0.89)
University country [d]				
England	215	79.34	824,835	81.85
Scotland	25	9.23	101,940	10.12
Wales	19	7.01	57,775	5.73
Northern Ireland	2	0.74	23,180	2.30
(Did not respond)	(10)**	(3.69)		

Note. Figures may not add up to 100% due to rounding.
[a] Due to HESA's (2019) data collection methods, some categories have been altered. [b] The HESA only collect ethnicity data for UK-domiciled students. [c] Only select HEIs collected data on highest level of educational attainment for the HESA. [d] The HESA do not collect data for the Open University, so this category was removed.
**$p < .01$
